# Evaluation of BoostDM, a somatic variant prediction tool, for the interpretation of germline variants in hereditary cancer genes

**DOI:** 10.1038/s41431-026-02077-y

**Published:** 2026-03-16

**Authors:** Elisabet Munté, Ferran Muiños, Raúl Marín, Lidia Feliubadaló, Federica Brando, Núria López-Bigas, Conxi Lázaro, Abel González-Pérez, Laura Valle

**Affiliations:** 1 Hereditary Cancer Program, Catalan Institute of Oncology; Oncobell Program, IDIBELL, Hospitalet de Llobregat, Barcelona, Spain; 2 Centro de Investigación Biomédica en Red en Cáncer (CIBERONC), Instituto de Salud Carlos III, Madrid, Spain; 3 Institute for Research in Biomedicine (IRB Barcelona), The Barcelona Institute of Science and Technology, Barcelona, Spain; 4 Department of Medicine and Life Sciences, Universitat Pompeu Fabra, Barcelona, Spain; 5 Institució Catalana de Recerca i Estudis Avançats (ICREA), Barcelona, Spain

**Keywords:** Cancer genetics, Cancer genomics, Cancer genetics

## Abstract

Classifying germline variants in hereditary cancer genes remains challenging and requires integrating diverse lines of evidence. BoostDM is a computational method originally developed to identify somatic cancer driver mutations by detecting signals of positive selection. Given the functional overlap between somatic and germline pathogenic variants in cancer genes, we evaluated the utility of BoostDM for interpreting germline variants in hereditary cancer genes. We assessed BoostDM’s performance across six genes with dual roles in sporadic and hereditary cancer (*ATM*, *BRCA1*, *BRCA2*, *CDH1*, *PTEN*, *TP53*), using gene-specific BoostDM models. A total of 1275 germline single nucleotide variants with expert-reviewed pathogenic and benign classifications were included. BoostDM scores were compared to those from AlphaMissense and REVEL, two leading pathogenicity predictors for missense variants. BoostDM correctly classified 74.5% of pathogenic/likely pathogenic and 98.6% of non-synonymous benign/likely benign variants overall. It performed particularly well for non-synonymous, non-missense variants (92.3% sensitivity). For missense variants, BoostDM correctly identified 46% of pathogenic and 95.5% of benign variants. While BoostDM did not outperform AlphaMissense or REVEL, it demonstrated high specificity (99.5%) and positive predictive value (PPV = 98%) for missense variants with high scores ( > 0.5). Gene-specific performance varied, with *TP53* showing the most robust results. In conclusion, BoostDM predictions are not a replacement for ACMG/AMP-guided germline variant classification, especially for missense changes. However, its high specificity and PPV suggest that high BoostDM scores can provide supportive evidence of pathogenicity, prompting further clinical and functional investigation.

## Introduction

Reaching a conclusive classification of germline variants in hereditary cancer genes is an arduous and challenging task that requires the integration of diverse types of evidence, including population allele frequencies, computational predictions of functional impact, phenotypic and family history information, and results from experimental functional studies. These are evaluated individually and translated into supporting, moderate, strong and stand-alone evidence, according to the standard ACMG/AMP variant classification guidelines [1], and whenever available, to gene-specific recommendations based on those guidelines (https://clinicalgenome.org/).

In this context, BoostDM emerges as a potentially valuable tool for informing variant interpretation. BoostDM is a computational method developed to classify somatic single-nucleotide variants according to their potential to drive tumorigenesis [2]. This tool is used to identify driver mutations from all somatic variants identified in a patient’s tumor. By learning from large tumor sequencing datasets, the method scores and classifies all possible single base substitutions based on a small set of features known to represent signals of positive selection. Boost DM models are specific of gene and tumor type.

The features incorporated into BoostDM models include the predicted consequence of the variant on the coding sequence (e.g., missense, nonsense, synonymous and potential splicing alterations), as determined by the Ensembl Variant Effect Predictor (VEP) [3]. Structural and functional relevance is further captured by the variant’s location within mutational clusters —identified in the three-dimensional (HotMaps3D) (ref. [4]) or linear protein structures (oncodriveCLUSTL) [5], or within recurrently mutated Pfam domains (smRegions) [6]. Nucleotide evolutionary conservation across vertebrates, assessed by PhyloP scores [7], is also included. For nonsense variants, BoostDM considers whether the premature stop codon is likely to trigger nonsense-mediated mRNA decay. Additional annotations include whether the variant alters residues involved in post-translational modifications—such as acetylation, methylation, phosphorylation, and ubiquitination—or affects known regulatory sites, as those annotated in PhosphoSitePlus [8]. BoostDM predictions are publicly accessible for all possible variants across specific cancer genes and tumor types at IntOGen https://www.intogen.org/boostdm/search.

Assuming that somatic cancer driver variants and germline pathogenic variants affecting the same cancer genes exert similar effects on protein function, we asked whether BoostDM, originally trained on somatic data, can accurately classify germline variants in hereditary cancer genes.

## Materials and methods

### BoostDM models and gene selection

Each boostDM model is a consensus aggregator of 50 individual gradient boosting classifiers (referred to as base models), each trained on a randomly selected, balanced subset of the training data. The aggregator produces a probabilistic prediction by combining the individual outputs of the base models and applying a correction for systematic bias. Intuitively, the systematic bias quantifies the extent to which base models regress towards log-odds zero due to partial information or under-confidence while issuing their respective forecasts. Each base model is trained with cross-entropy loss and early stopping. The decision trees are restricted to a maximum depth of 4. For more detailed information, please refer to the method’s documentation: https://boostdm.readthedocs.io/en/latest/method_closeup.html.”

A model is trained for each driver gene (informed by IntOGen) and a tumor type, meaning that the variants mapping to the gene are observed in samples matching the tumor type, thereby reflecting the propensities that are specific for that gene in that tumor type. For a given driver gene, models are trained across all tumor types in a hierarchical ontology, ranging from the broadest category (CANCER) down to the specific tumor types in which IntOGen identified the gene as a cancer driver, including all intermediate categories. For example, lung adenocarcinoma (LUAD) and lung squamous cell carcinoma (LUSC) are both subtypes of non-small cell lung cancer (NSCLC), which in turn belongs to solid tumors (SOLID), itself a subset of CANCER. Accordingly, a SOLID-level model for a given gene was trained using variants mapping to that gene observed in samples from any solid tumor type in which the gene is considered a cancer driver.

Because the richness of variant catalogs varies across genes and tumor types, it is not always possible to successfully train a model for a specific gene–tumor type combination. In such cases, using a more general tumor category allows the aggregation of a sufficiently large training set for the model to achieve a minimum acceptable level of performance. At the same time, models trained on broader tumor categories, such as SOLID, can capture positive selection propensities that are shared across the different tumor subtypes included within that category.

BoostDM v.2024-09.20-cancer data were downloaded from the IntOGen website (https://www.intogen.org/boostdm/downloads). From the available genes with BoostDM models (Supplemantry Table 1), *ATM, BRCA1, BRCA2, CDH1, PTEN*, and *TP53* were selected for this study based on their dual role as both cancer driver genes and hereditary cancer predisposition genes, and the availability of >100 unique single base substitutions classified as pathogenic, likely pathogenic, benign or likely benign considering ClinGen and our in-house variant dataset.

For each gene, the BoostDM models trained on cancer types within the tumor spectrum associated with the corresponding hereditary syndrome were considered. These were called “phenotype-specific models”. When no such phenotype-specific model was available, the model trained on generic “SOLID cancers” was used. As mentioned before, a “SOLID cancer model” refers to a model based on mutations observed in a particular gene across solid tumor types for which that gene is considered driver according to IntOGen. For example, the SOLID cancer model for PTEN is based on mutations identified in breast (BRCA) and colorectal (COREAD) cancers. Models labeled as BRCA, COAD, GBM or LUAD refer to models trained on specific cancer types (e.g., BRCA for invasive breast carcinoma, COAD for colon adenocarcinoma, GBM for glioblastoma multiforme, LUAD for lung adenocarcinoma).

In the case of *ATM*, neither a matching phenotype-specific nor a SOLID cancer model was available. Of the three models available (all lung cancer subtypes), the lung adenocarcinoma (LUAD) model was selected.

### Pathogenic and benign variants included in the study

Pathogenic and benign single nucleotide variants (single base substitutions) in the six genes of interest were obtained from the ClinGen Evidence Repository (n = 447 variants; https://erepo.genome.network/evrepo/, accessed July 2024). An additional in-house dataset comprising 1,032 single base substitutions was also included. This dataset consisted of variants classified by diagnostics experts according to ClinGen gene-specific guidelines (up to January 2025), along with benign or likely benign variants previously evaluated using general ACMG/AMP criteria. The latter were re-evaluated using vaRHC [9], a semi-automated classification tool that applies gene-specific rules; only variants meeting the BA1 or BS1 criteria were retained. Variants of uncertain significance were excluded from analysis. Overlapping variants between ClinGen and in-house datasets (n = 66) were counted only once; in all cases, there was 100% concordance in classification.

To enable integration with the BoostDM dataset, variant genomic coordinates were converted from GRCh37 to GRCh38 using the rtracklayer package in the R environment (v4.2.1) (ref. [10]). Only variants for which a BoostDM score was available were retained. The final dataset consisted of 1275 single base substitutions.

### Variant annotation and in silico predictors

Variants were annotated using the Ensembl Variant Effect Predictor [3] (VEP v.113) based on the MANE Select transcript [11]. Pathogenicity scores, including REVEL [12] and AlphaMissense [13, 14], were obtained using VEP from the corresponding raw data files. For all three predictors, pathogenicity scores range from 0 to 1, with 1 indicating the highest probability of pathogenicity. The pathogenicity threshold for BoostDM was set to >0.5, as recommended by its developers. No pathogenicity thresholds were applied to REVEL or AlphaMissense scores.

The presence of variants in the TCGA and COSMIC databases (somatic variants) was annotated by retrieving data from the UCSC Table Browser [15] (TCGA Pan-Cancer and COSMIC v101 tracks).

The complete list of variants included in the analysis, along with their annotations, HGVS nomenclature, and pathogenicity scores, is provided in Supplementary Table 2.

### Data analysis

Data processing and visualizations were performed using R (v.4.2.1). Performance metrics (accuracy, sensitivity, specificity, positive predictive value (PPV), negative predictive value (NPV) and F1 score) for missense variants were calculated using the caret R package (v.7.0.1). Area under the ROC curve (AUC) values for BoostDM, AlphaMissense and REVEL were computed with the yardstick R package (v 1.3.2). Pair-wise differences between AUCs were assessed with the DeLong’s test implemented in pROC (v 1.18.5) [16].

## Results and discussion

We selected genes that are recognized both as somatic drivers in cancer, and as causative genes in hereditary cancer syndromes, for which BoostDM models are available (Supplementary Table 1) and with >100 unique single base substitutions classified as pathogenic, likely pathogenic, benign or likely benign considering ClinGen and our in-house variant dataset. These included *ATM*, *BRCA1, BRCA2, CDH1, PTEN* and *TP53*. For each gene, we utilized BoostDM models trained on cancer types that correspond to the organs primarily affected in the tumor spectrum of the associated hereditary syndrome [17–24]. When not available, we used models trained on “SOLID cancers”, or other available (Table 1**)**.

**Table 1:** Genes evaluated, cancer-specific BoostDM models used for classification, number of variants evaluated, and performance of best-performing models.

Gene	Main cancers associated with the hereditary cancer syndrome	No. of cancer types BoostDM is trained for	BoostDM cancer-specifc models used^a^	Variants evaluated				Variants correctly classified using BoostDM			
				(L)P		(L)B		(L)P		(L)B	
*ATM*	Breast, prostate, pancreas, stomach	3	**LUAD**^**b**^	84		129		68 (80.95%)		129 (100%)	
*BRCA1*	Breast, ovary, prostate, pancreas	2	**SOLID**	112		150		64 (57.14%)		150 (100%)	
*BRCA2*	Breast, ovary, prostate, pancreas	2	**SOLID**	94		254		65 (69.15%)		252 (99.21%)	
*CDH1*	Stomach, breast	4	**BRCA** SOLID	81		101		74 (91.36%)		98 (97.03%)	
*PTEN*	Breast, thyroid, kidney, uterus, colorectum	7	**SOLID**	96		16		63 (65.63%)		16 (100%)	
*TP53*	Sarcoma, breast, brain, leukemia, colorectum, lung, adrenocortical tumors	56	**BRCA** COAD GBM LUAD	95		63		85 (89.47%)		61 (96.83%)	
TOTAL (n = 1275)				562		713		419 (74.55%)		706	
TOTAL excluding synonymous variants (n = 986)				562		424		419 (74.55%)		418	

^a^In bold, best performing model. *BRCA* invasive breast carcinoma, *COAD* colon adenocarcinoma, *GBM* glioblastoma multiforme, *LUAD* lung adenocarcinoma, *SOLID* solid cancer.

^b^All three available BoostDM models for ATM were trained with lung cancer subtypes, which do not belong to the tumor spectrum of the ATM-associated hereditary cancer syndrome.

*(L)B* benign or likely benign, *(L)P* pathogenic or likely pathogenic.

A total of 1275 germline single base substitutions in the selected genes (*ATM*, *BRCA1*, *BRCA2*, *CDH1*, *PTEN*, and *TP53*) were included in the analysis. These variants had been previously classified as pathogenic or likely pathogenic (hereafter referred to as PVs; *n* = 562), and benign or likely benign (hereafter referred to as BVs; *n* = 424) based on ACMG/AMP variant classification guidelines, as curated by ClinGen expert panels (source: https://erepo.clinicalgenome.org/evrepo/) or by the Genetic Diagnostic Unit of the Hereditary Cancer Program at the Catalan Institute of Oncology (Table 1). Of the total PVs and BVs analyzed, 41.5% (529/1,275) were also reported as somatic variants in public cancer datasets (TCGA and/or COSMIC). When considering only PVs, this proportion increased to 54% (302/562), highlighting the substantial overlap between germline PVs and somatic alterations in tumors. The complete list of germline variants analyzed, including their pathogenicity scores and presence in TCGA or COSMIC, is provided in Supplementary Table 2.

Applying BoostDM’s established threshold for the categorization of somatic variants —categorizing variants with a score > 0.5 as likely driver mutations and those with a score ≤ 0.5 as likely passenger mutations— the best cancer type-specific models for each gene correctly classified 74.5% (419/562) of PVs and 99% (706/713) of all BVs, or 98.6% (418/424) of non-synonymous BVs.

When analyzed by gene, performance varied. For *ATM*, using the BoostDM model trained on lung adenocarcinoma data, 80.9% (68/84) of PVs were identified as likely drivers, while all BVs (129/129) were correctly classified as non-drivers. Because phenotype-matched/SOLID models were not available for *ATM*, it was analyzed using a non–phenotype-matched model (lung adenocarcinoma); therefore, the corresponding results should be interpreted with caution. For *BRCA1* and *BRCA2*, the SOLID cancer model correctly classified 57.1% (64/112) and 69.1% (65/94) of PVs, and 100% (150/150) and 99.2% (252/254) of BVs, respectively. The *CDH1*, *PTEN*, and *TP53* models —trained on breast, SOLID, and breast cancer data, respectively— achieved correct classification rates of 91.4% (74/81), 65.6% (63/96), and 89.5% (85/95) for PVs, and 97.0% (98/101), 100% (16/16), and 96.8% (61/63) for BVs (Table 1). The performance of additional gene–cancer model combinations is detailed in Supplementary Table 3.

**Fig. 1:**
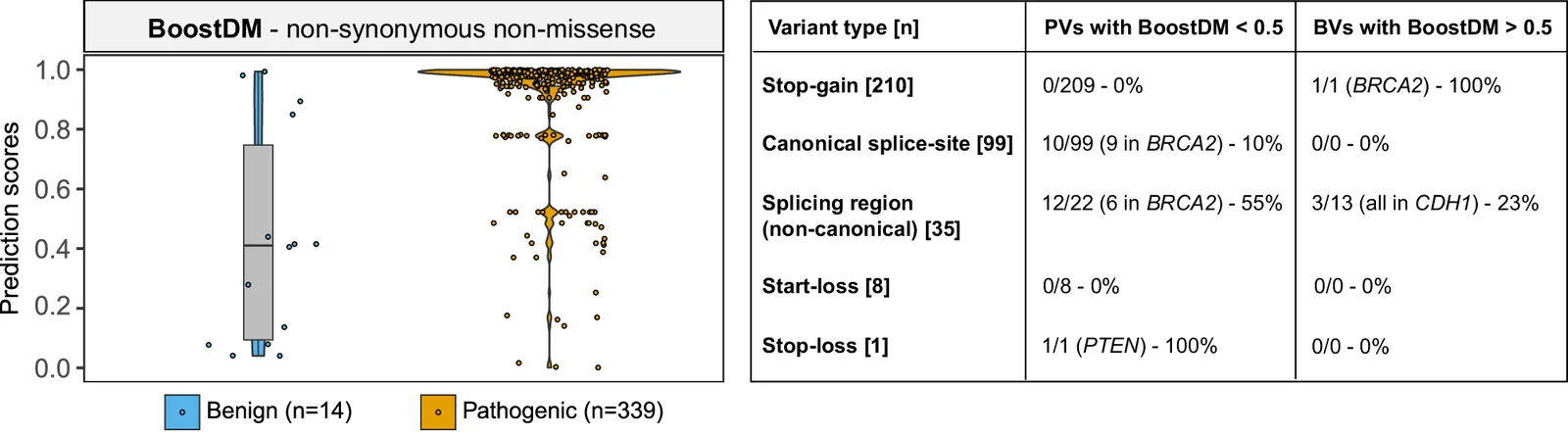
BoostDM scores for non-synonymous, non-missense variants and correspondence with ACMG/AMP-based classifications according to variant type. Left panel: Distribution (box and violin plots) of BoostDM scores for non-synonymous, non-missense pathogenic and benign variants. Right panel: Type of variants and BoostDM misclassifications by variant type.

BoostDM showed robust classification performance for non-synonymous non-missense variants —including stop-gain, start-loss, stop-loss, and splicing-region variants—, correctly classifying 92.3% (326/353) of them (Fig. 1). Notably, the majority of misclassified variants in this category (25 of 27) were splicing-region variants affecting both canonical and non-canonical splicing positions. Most incorrectly classified splicing-related PVs were observed in *BRCA2*, accounting for 68% (15/22) of all splicing PV misclassifications (Supplemantry Fig. 1). A plausible explanation for this observation is that in the IntOGen pipeline, splicing mutations in *BRCA2* do not show a sufficiently strong positive selection signal, as estimated by splicing-specific dN/dS values —a metric that quantifies whether splicing-disrupting mutations occur more often than expected by chance—to meet the threshold for inclusion in the positive training set. As a result, most splicing-affecting variants are excluded during model training, reducing the contribution of splicing-related features to the final classifier. This can be an issue for cancer driver genes in which splicing variants contribute significantly to pathogenicity but the splicing positive selection signal is not strong enough. One possible avenue to address this limitation would be to relax the criteria for including mutations in the training set.

**Fig. 2:**
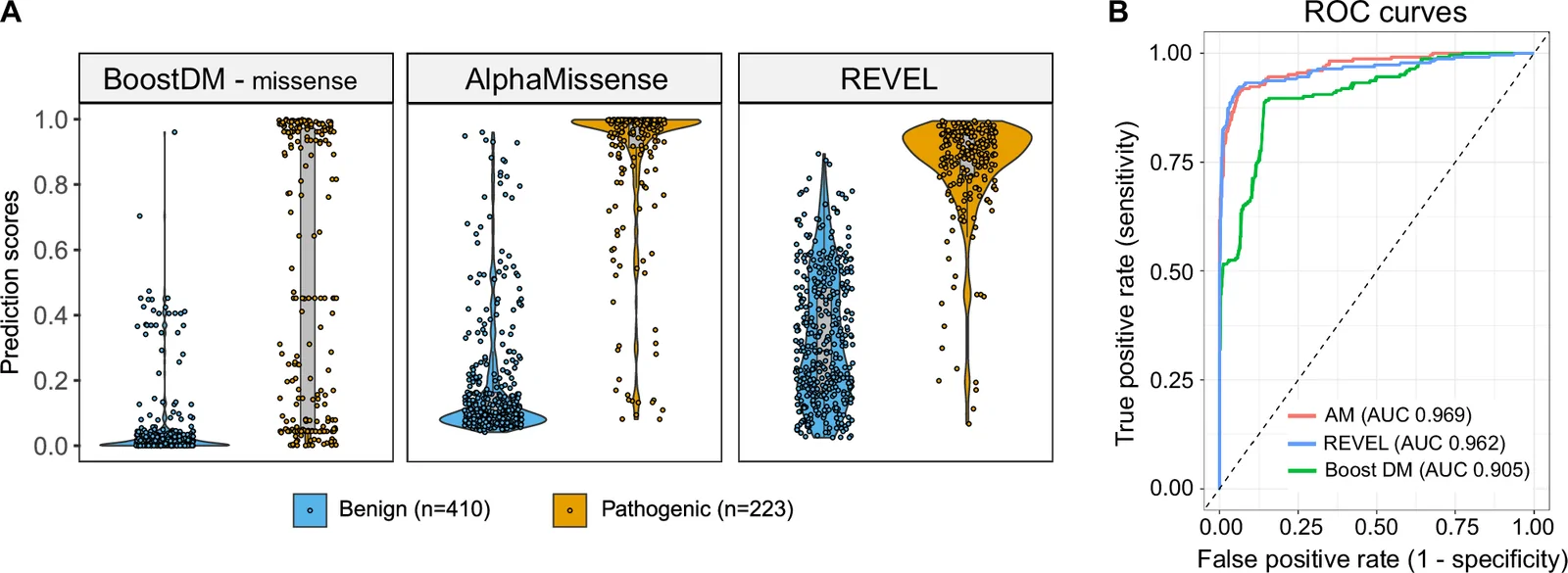
Performance of BoostDM, AlphaMissense and REVEL scores for the prediction of pathogenic and benign missense variants in the studied genes. **A** Distribution (box and violin plots) of scores for benign and pathogenic variants. **B** Receiver operating characteristic (ROC) curves and area under the curve (AUC) values for each predictor.

As expected, the performance of BoostDM for missense variants was lower than for loss-of-function variants. BoostDM correctly classified 46% (103/223) of missense PVs and 99.5% (408/410) of missense BVs (Fig. 2). Unfortunately, over 50% of PVs received a benign BoostDM score, with misclassifications affecting all genes (Supplemantry Table 2, Supplemantry Fig. 2).

We compared BoostDM to AlphaMissense [14] and REVEL [12] —two high-performing, ClinGen-endorsed tools for missense variant pathogenicity prediction [25]— in their ability to classify germline missense variants. The continuous output scores (ranging from 0 to 1 for all predictors), without any binary categorization, were used to compare the performance of the tools. While BoostDM demonstrated competitive performance (AUC = 0.905; 95% CI: 0.881–0.930), it did not outperform AlphaMissense (AUC = 0.969; 95% CI: 0.955–0.982) or REVEL (AUC = 0.962; 95% CI: 0.944–0.980) in overall pathogenicity prediction accuracy (Fig. 2A and B). Pairwise AUC comparisons using DeLong’s test revealed significant differences between BoostDM and AlphaMissense (*p* = 1.45×10⁻⁷), and between BoostDM and REVEL (*p* = 1.46×10⁻⁵), but not between AlphaMissense and REVEL (*p* = 0.355).

Importantly, a low BoostDM score ( ≤ 0.5) was not a reliable indicator of benignity, as 23% (120/528) of missense variants predicted as benign by BoostDM were classified as PVs by ClinGen or expert review. In contrast, a high BoostDM score ( > 0.5) was strongly predictive of pathogenicity: 98% of these missense variants were confirmed as PVs. These findings suggest that, although BoostDM may have limited utility for excluding pathogenicity, a high score could serve as supportive evidence for pathogenicity (consistent with ACMG/AMP rule PP3) when interpreting missense variants. Interestingly, only missense variants in *TP53* and *PTEN* received BoostDM scores >0.5 (Supplementary Fig. 2).

To further evaluate the gene-specific performance of BoostDM for missense variant classification, we calculated key performance metrics for each gene and across all genes (Table 2). While BoostDM models consistently achieved high specificity, sensitivity varied considerably by gene. Notably, BoostDM failed to identify any pathogenic missense variants in *ATM*, *BRCA1*, *BRCA2*, and *CDH1*, resulting in a sensitivity of 0.000 for these genes —indicating that all PVs were incorrectly classified as benign at the standard >0.5 threshold. The *PTEN* model demonstrated moderate sensitivity (0.523) and perfect specificity (1.000), yielding a positive predictive value (PPV) of 1.000 but a low negative predictive value (NPV = 0.139), underscoring a high false-negative rate for benign predictions. The *TP53* model showed the best performance, with high sensitivity (0.885), specificity (0.964), and F1 score (0.926), indicating that the BoostDM model for *TP53* is particularly effective for distinguishing pathogenic from benign missense variants. The reasons for the observed inter-gene variability in BoostDM performance for missense variants remain unclear and warrant further investigation, particularly as additional classified variants become available for genes with smaller numbers of missense PVs, such as *ATM*, *BRCA2*, and *CDH1*.

**Table 2:** Performance metrics of BoostDM for missense variant pathogenicity prediction per gene, applying the 0.5 score threshold.

Gene	Accuracy	Sensitivity	Specificity	PPV	NPV	F1
*ATM*	0.776	0.000	1.000	NA	0.776	NA
*BRCA1*	0.656	0.000	1.000	NA	0.656	NA
*BRCA2*	0.910	0.000	1.000	NA	0.910	NA
*CDH1*	0.910	0.000	1.000	NA	0.910	NA
*PTEN*	0.557	0.523	1.000	1.000	0.139	0.687
*TP53*	0.918	0.885	0.964	0.972	0.857	0.926
**All genes**	0.807	0.462	0.995	0.981	0.773	0.628

*PPV* positive predictive value, *NPV* negative predictive value, *F1* F1-score, the harmonic mean of precision and sensitivity. NA indicates insufficient data to calculate the metric.

We acknowledge the potential limitations associated with using BoostDM models trained on cancer types that may not be directly relevant to the corresponding hereditary syndrome (e.g., lung adenocarcinoma for *ATM* or generalized “SOLID cancer” models for other genes). Additionally, there may be limitations stemming from the assumption that the mechanisms underlying somatic mutations in tumor cells are similar to those driving germline variants. In particular, germline PVs are often not present in somatic datasets, as expected, due to the positive selection processes that drive somatic mutations in tumors. Somatic cancer drivers are selected for their ability to confer growth advantages to the tumor, while germline variants may have different biological effects that do not confer such selective advantages. This discrepancy may influence the accuracy of somatic model predictions when applied to germline contexts. Further research and the development of models specifically trained on germline variant data would be beneficial to improve the accuracy of pathogenicity predictions in hereditary contexts.

## Conclusions

Our results indicate that BoostDM —and, by extension, likely other machine learning models trained on signals of positive selection to identify somatic driver mutations— cannot currently replace ACMG/AMP guideline-based classification for germline variants in hereditary cancer genes. Ideally, computational models should be specifically developed for germline variant interpretation in this context. However, the limited number of germline variants, especially missense variants, classified as (likely) pathogenic or (likely) benign under ACMG/AMP criteria in rare hereditary cancer genes significantly hampers the feasibility of building robust gene-specific models. Surpassing the ACMG/AMP framework —including its increasingly refined gene-specific specifications— remains a major challenge, given its comprehensive and integrative approach that incorporates population data, in silico predictions, clinical and tumor characteristics, family history, and functional evidence.

BoostDM generally does not outperform leading in silico tools such as AlphaMissense or REVEL in terms of predictive power. However, it offers key advantages: it generates predictions for both missense and non-missense variants, and, most notably, a high BoostDM score ( > 0.5) is a strong indicator of pathogenicity for missense variants, with an overall positive predictive value of 0.981. As such, the identification of a variant of uncertain significance with BoostDM score >0.5 should prompt additional investigations such as extended co-segregation analyses, functional assays, and/or tumor studies, in order to achieve a clinically meaningful classification. Whether the observations from our study, which focused on six specific genes, can be generalized to other hereditary cancer genes remains to be determined, particularly in light of the observed variability in inter-gene performance of the tool.

## Supplementary information

Supplementary Figs. 1 and 2

Supplementary Tables 1, 2 and 3

## Data Availability

The datasets (variants) analyzed during the current study are available in https://erepo.genome.network/evrepo/ (ClinGen curated variants) and in a private (in-house) database. In silico predictions (BoostDM, REVEL and AlphaMissense scores) may be obtained from: https://www.intogen.org/boostdm/downloads; https://doi.org/10.5281/zenodo.10813168; https://doi.org/10.5281/zenodo.7072866. All data used in this article are included in the text or supplementary materials.
