# Supplementary material for: Evaluation of BoostDM, a somatic variant prediction tool, for the interpretation of germline variants in hereditary cancer genes
Source: Eur J Hum Genet. 2026 Mar 16;34(9):1336–41. doi: 10.1038/s41431-026-02077-y (PMC13550587; doi:10.1038/s41431-026-02077-y)
Supplement: Supplementary file 1 [file 41431_2026_2077_MOESM1_ESM.pdf]

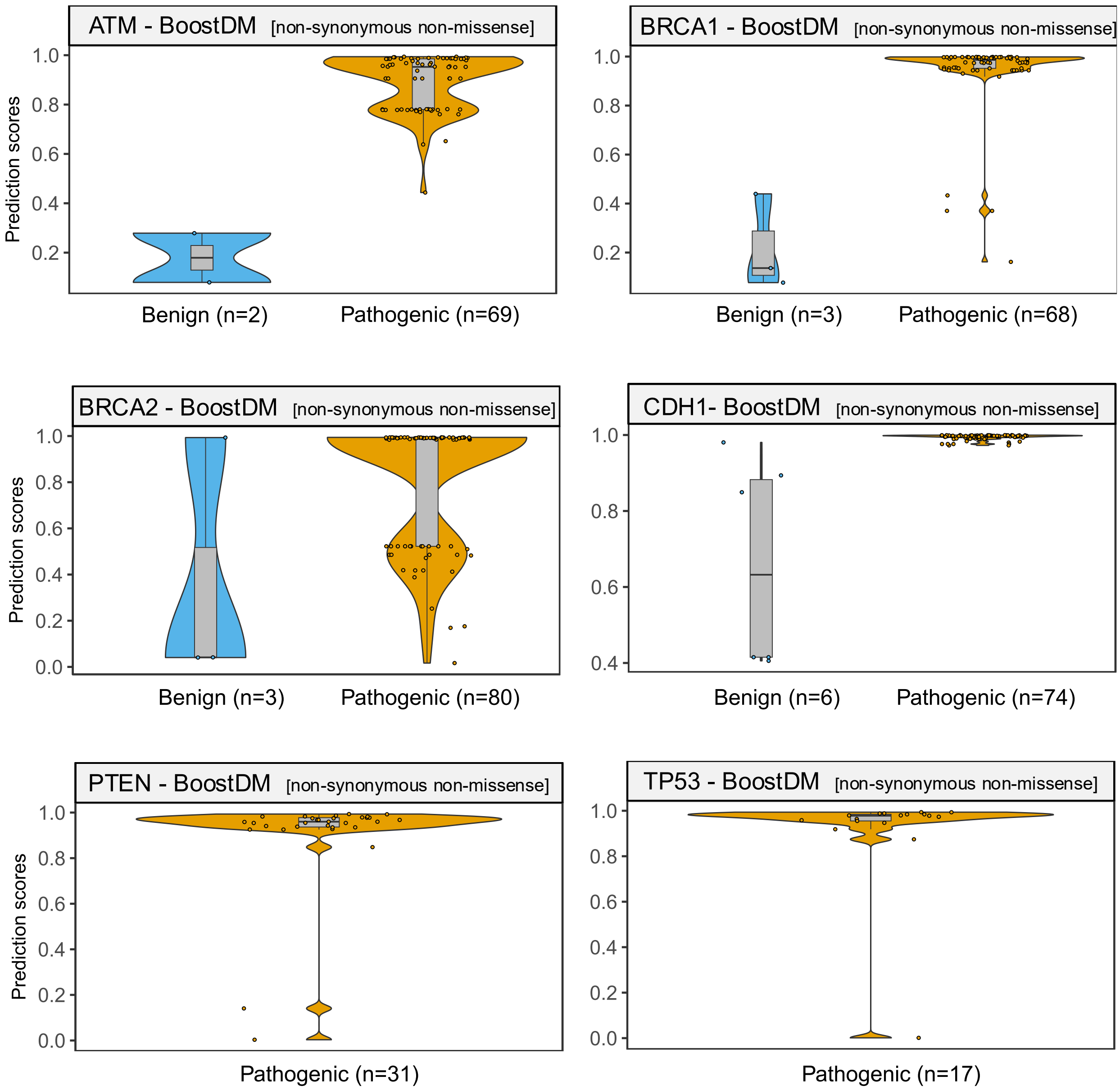

Supplementary Figure 1. Distribution (box and violin plots) of BoostDM scores for non-synonymous, non-missense pathogenic and benign variants in *ATM*, *BRCA1*, *BRCA2*, *CDH1*, *PTEN* and *TP53*.

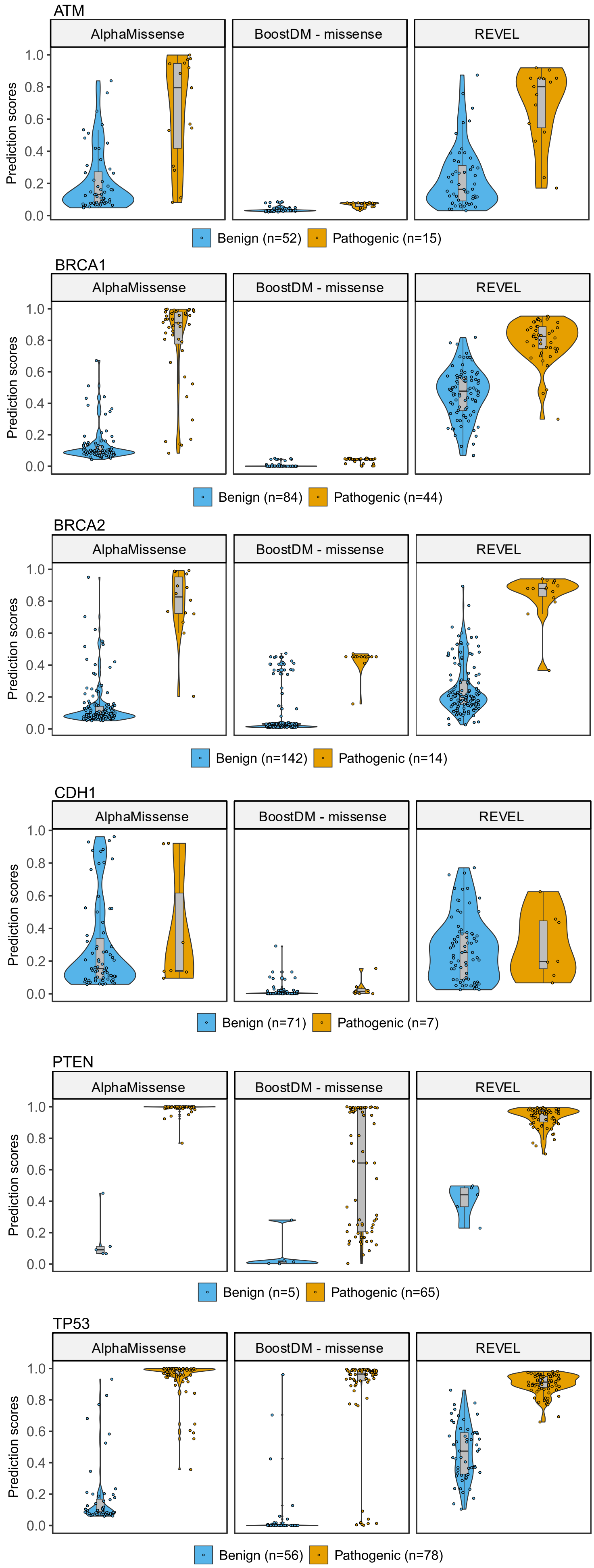

Supplementary Figure 2. Distribution (box and violin plots) of AlphaMissense, BoostDM, and REVEL scores for missense pathogenic and benign variants in *ATM*, *BRCA1*, *BRCA2*, *CDH1*, *PTEN* and *TP53*.
